# AP-3 shows off its flexibility for the cryo-EM camera

**DOI:** 10.1016/j.jbc.2021.101491

**Published:** 2021-12-10

**Authors:** Todd R. Graham

**Affiliations:** Department of Biological Sciences, Vanderbilt University, Nashville, Tennessee, USA

**Keywords:** AP-3, AP-1, AP-2, clathrin, cryo-EM, phosphoinositide, ARF, Golgi, AP, adaptor protein, ARF, ADP-ribosylation factor

## Abstract

The tetrameric adaptor protein AP-3 is critical for the transport of proteins to lysosomes and lysosome-related organelles. The structures of homologous adaptors AP-1 and AP-2 have revealed a closed-to-open conformational change upon membrane recruitment and phosphoinositide binding. Recently, Schoppe *et al.* reported the first cryo-EM structures of AP-3 from budding yeast and described remarkably flexible solution structures that are all in the open conformation. The apparent lack of a closed conformational state, the first such description in the literature, allows AP-3 to be more reliant on cargo interaction for its initial membrane recruitment compared with AP-1.

The formation of membrane-enclosed transport vesicles (or tubules) typically requires coat proteins such as clathrin to select cargo and facilitate membrane deformation. Clathrin was the first identified vesicle coat protein and associates with a variety of different adaptor proteins (APs) that bind to sorting signals found in cargo proteins (1, 2). These APs can be differentially recruited to the Golgi, endosomes, or plasma membrane, providing clathrin the versatility of operating in several different trafficking pathways. The five known tetrameric adaptors, termed AP-1 to AP-5, have similar subunit compositions, with two large subunits bearing long-unstructured linkers attached to “ear” domains (β1–5 and α, γ, δ, ε, or ζ), a medium-sized subunit (μ1–5), and a small subunit (σ1–5). For AP-3, the subunit composition is β3, δ, μ3, and σ3 (2). These APs are well conserved, although AP-4 and AP-5 are not present in many model organisms, such as budding yeast and *Drosophila*.

The role AP-3 plays in protein trafficking was illuminated through the characterization of mutations causing changes in eye color in *Drosophila* (*garnet*), coat color in mice (*mocha*, *pearl*), and mislocalization of alkaline phosphatase in yeast (3). Soon after these reports, AP-3 mutations in humans were found to cause Hermansky–Pudlak syndrome, which is characterized by albinism, defects in blood clotting, immunodeficiency, and pulmonary fibrosis. These studies emphasized the importance of AP-3 in sorting proteins to lysosome-like organelles, including melanosomes, platelet-dense granules, lamellar bodies, and the yeast vacuole (4, 5). In budding yeast, AP-3 has been shown to mediate a direct Golgi-to-vacuole transport pathway that bypasses the endosomes (6). Another striking feature of budding yeast AP-3 is that it functions quite well in the absence of clathrin, which may be true for mammalian AP-3 as well (2, 6). The clathrin outer coat formed from triskelia of heavy and light chains is thought to be important for concentrating cargo and deforming the membrane. However, how AP-3 sorts proteins without this clathrin functionality during vesicle budding is unclear.

All of the tetrameric adaptors recognize similar YXXΦ and dileucine sorting signals in cargo proteins and, therefore, must have other binding interactions that confer organelle-specific membrane recruitment (1). AP-1, AP-3, and AP-4 all require the small GTP-binding protein ADP-ribosylation factor (ARF) in its GTP-bound form for recruitment to Golgi or endosomal membranes. There also appears to be a general requirement for anionic phospholipid, particularly phosphoinositides, for membrane association. For example, AP-2 is recruited from the cytosol to the plasma membrane by binding phosphatidylinositol-4,5-bisphosphate and sorting signals, whereas AP-1 is recruited to the *trans*-Golgi network by binding to PI4P, Arf-GTP, and sorting signals present in integral membrane cargo proteins (2, 7, 8).

Both AP-1 and AP-2 adopt a closed conformational state when free in solution. This conformation buries the cargo-binding sites in the μ subunits, thereby preventing cargo engagement at inappropriate membrane sites (7, 8). Arf-GTP and/or phosphoinositide interactions induce a conformational change to an open state that swings the μ subunit into a position that allows cargo engagement (Fig. 1). Therefore, it seemed likely that the other tetrameric adaptors may be regulated in a similar fashion. However, although Schoppe *et al.* find here that AP-3 is highly flexible and can adopt a suite of open conformational states in solution, they did not detect the closed conformational state (9). If AP-3 does not rely on activating ligands to achieve the open state, then it should be able to bind cargo in the absence of Arf and phosphoinositides. Indeed, the authors found substantial recruitment of AP-3 to liposomes bearing cargo but lacking Arf and PI4P, whereas, as expected, AP-1 was not recruited onto the membrane under these conditions.

**Figure 1:**
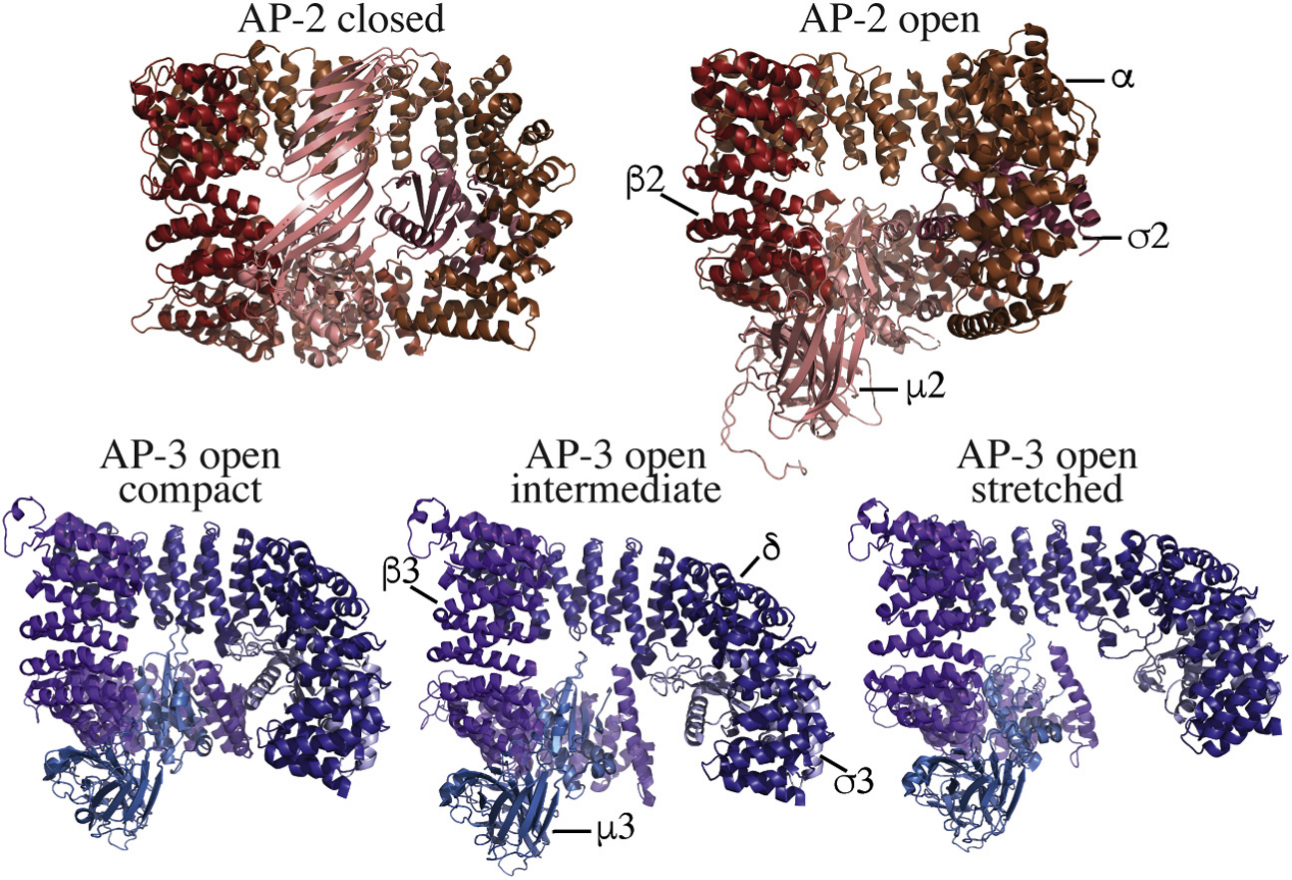
**AP-2 and AP-3 conformational states.** AP-2 primarily exists in a closed conformational state (PDB, 2VGL) in solution and undergoes a conformational switch to the open structure (PDB, 2XA7) upon binding to phosphoinositides present in the inner leaflet of the plasma membrane. In these views, the μ2 subunit swings downward from the AP-2 core during activation to expose cargo-binding sites. In contrast, purified AP-3 is only detected in the open conformation by cryo-EM but with conformational states ranging from open compact (PDB, 7P3X), open intermediate (PDB, 7P3Y), and open stretched (PDB, 7P3Z). The AP structures shown lack the ear appendages.

However, AP-3 binding to sorting signals in cargo does not appear to be sufficient for stable membrane association, otherwise AP-3 would be recruited to newly synthesized cargo in the ER and *cis*-Golgi. Within living yeast cells, AP-3 is recruited to maturing Golgi cisternae in the early Golgi-to-late Golgi transition, before appearance of the canonical late Golgi marker Sec7 and AP-1 binding (10). Therefore, it was hypothesized that PI4P would play a role in AP-3 membrane recruitment and consistently Schoppe *et al.* (9) found that PI4P acts with Arf-GTP to stimulate AP-3 binding to liposomes. However, through these direct comparisons, it appears that cargo interactions play a much greater role in AP-3 membrane recruitment relative to AP-1 membrane recruitment (9).

These results provide an elegant explanation for why AP-3 precedes AP-1 on maturing Golgi cisternae (9). The lack of a closed conformational state should make AP-3 a more sensitive coincidence detector, as it can immediately bind cargo, Arf-GTP, and PI4P without having to undergo a ligand-induced conformational change. Assuming PI4P is limiting in the membrane during the early-to-late Golgi transition, AP-3 should be able to outcompete AP-1 for these initial interactions because AP-1 would not have a comparable avidity for the membrane while in the closed conformational state. As AP-3 cargoes are removed and PI4P levels increase, AP-1 would then have sufficient access to Arf-GTP and PI4P to undergo the conformational change needed for cargo binding, high avidity membrane association, and vesicle formation. The study by Schoppe *et al.* (9) also provides hints as to how AP-3 can concentrate cargo in the absence of clathrin. In solution, AP-3 fractionates as a single, well-defined peak by gel filtration, indicating a lack of oligomerization. However, upon recruitment of an equimolar mixture of differentially labeled AP-3s to supported bilayers containing cargo, dually labeled aggregates of low diffusional mobility were formed (9). This observation suggests that cargo binding on a membrane surface can promote AP-3 self-assembly, thereby concentrating cargo. Thus, the ability of clathrin to cross-link adaptors in a defined region of the membrane would not be needed. An exciting future direction would be to define the molecular basis for oligomerization and test whether these AP-3 self-interactions are critical for cargo sorting to the vacuole.

Although these biochemical studies clearly demonstrate fascinating differences between AP-1 and AP-3 (9), it remains possible that an AP-3 closed conformational state exists in the cytosol of cells and is stabilized by factors lost upon its purification. Coat function can be modulated by posttranslational modification, and even though AP-3 was purified from yeast, it is difficult to know whether the modifications are gained or lost during cell lysis and protein purification. The development of *in vivo* detectors of conformational changes would provide a complementary approach to compare AP-1 and AP-3 dynamics in living cells.

## Conflict of interest

The author declares that he has no conflict of interest with the contents of this article.
